# Assessing the Relative Value of CT Perfusion Compared to Non-contrast CT and CT Angiography in Prognosticating Reperfusion-Eligible Acute Ischemic Stroke Patients

**DOI:** 10.3389/fneur.2021.736768

**Published:** 2021-09-09

**Authors:** Andrew Bivard, Christopher Levi, Longting Lin, Xin Cheng, Richard Aviv, Neil J. Spratt, Tim Kleinig, Kenneth Butcher, Chushuang Chen, Qiang Dong, Mark Parsons

**Affiliations:** ^1^Melbourne Brain Centre, Royal Melbourne Hospital, University of Melbourne, Melbourne, VIC, Australia; ^2^Hunter Medical Research Institute, University of Newcastle, Newcastle, NSW, Australia; ^3^Department of Neurology, John Hunter Hospital, Faculty of Health, University of Newcastle, Newcastle, NSW, Australia; ^4^Department of Neurology, Liverpool Hospital, Liverpool, NSW, Australia; ^5^Ingham Institute for Applied Medical Research, Southwestern Sydney Clinical School, University of New South Wales, Liverpool, NSW, Australia; ^6^Department of Neurology, Huashan Hospital, Fudan University, Shanghai, China; ^7^Department of Medical Imaging, Sunnybrook Health Sciences Centre, University of Toronto, Toronto, ON, Canada; ^8^Department of Neurology, Royal Adelaide Hospital, Adelaide, SA, Australia; ^9^Prince of Wales Clinical School, University of New South Wales, Sydney, NSW, Australia

**Keywords:** reperfusion, brain imaging, computed tomography angiography, CT perfusion, ischemic stroke

## Abstract

In the present study we sought to measure the relative statistical value of various multimodal CT protocols at identifying treatment responsiveness in patients being considered for thrombolysis. We used a prospectively collected cohort of acute ischemic stroke patients being assessed for IV-alteplase, who had CT-perfusion (CTP) and CT-angiography (CTA) before a treatment decision. Linear regression and receiver operator characteristic curve analysis were performed to measure the prognostic value of models incorporating each imaging modality. One thousand five hundred and sixty-two sub-4.5 h ischemic stroke patients were included in this study. A model including clinical variables, alteplase treatment, and NCCT ASPECTS was weak (*R*^2^ 0.067, *P* < 0.001, AUC 0.605) at predicting 90 day mRS. A second model, including dynamic CTA variables (collateral grade, occlusion severity) showed better predictive accuracy for patient outcome (*R*^2^ 0.381, *P* < 0.001, AUC 0.781). A third model incorporating CTP variables showed very high predictive accuracy (*R*^2^ 0.488, *P* < 0.001, AUC 0.899). Combining all three imaging modalities variables also showed good predictive accuracy for outcome but did not improve on the CTP model (*R*^2^ 0.439, *P* < 0.001, AUC 0.825). CT perfusion predicts patient outcomes from alteplase therapy more accurately than models incorporating NCCT and/or CT angiography. This data has implications for artificial intelligence or machine learning models.

## Introduction

The pivotal intravenous thrombolysis trials, predominantly conducted more than a decade ago, did not use advanced imaging techniques such as perfusion imaging to identify a treatment target. Consequently, these trials provide limited guidance to the treating clinician when faced with the individual decision making (1). However, acute stroke treatment guided by more sophisticated imaging is being increasingly adopted in routine clinical practice following the positive endovascular therapy trials where the treatment targets were identified with CT angiography ± CT perfusion (2–4). Modern imaging not only identifies treatment targets such as a vessel occlusion and penumbral tissue (5, 6), but can also allow clinicians to develop more sophisticated risk-benefit judgements at the individual patient level. Given recent evidence, the debate regarding imaging of vessels and tissue perfusion has evolved; initially, from whether it should be performed at all in routine practice, and now, to which modern imaging protocols provide the best balance between the decision support information provided and the time taken to acquire and interpret the data. CT angiography was a minimum requirement of the recent endovascular trials in order to identify an occluded vessel and to assess vascular access in patients being considered for endovascular therapy. This technique is ideal for identifying large vessel occlusions, although more advanced (multiple time point) CTA may be necessary to provide additional accuracy in grading occlusion severity (partial or complete) and for identifying collateral blood flow beyond an occlusion. However, CTA does not accurately identify patients with penumbral tissue, and is poor at identifying the infarct core (7). Therefore, “limited” multimodal CT protocols only using NCCT and CTA may lead to futile reperfusion treatment where there is no penumbra to salvage and/or when there is an established infarct core with minimal change on NCCT, which is common (8). Perfusion imaging can positively identify the ischemic core and penumbra, and is now well-validated at identifying patients most likely to benefit from reperfusion. However, perfusion imaging does not identify a vessel occlusion (unless simultaneous dynamic CTA is acquired). However, CTP can quantitatively estimate collateral blood flow by measuring the severity of the perfusion deficit (9). Currently there is a lack of data comparing the usefulness of the various imaging approaches in use to predict outcome of patients treated with intravenous thrombolysis.

The individual patient response to reperfusion treatment is well-documented in ischemic stroke, and modern imaging can identify subgroups of patients with varying treatment responses. As such, there is an enormous potential for an AI based decision support system to be accurate, fast and widely available, if it is shown to be consistently reliable or related to individual patient outcomes. Importantly, the data which is used to generate a prediction for decision support needs to be relevant to the underlying disease process. This highlights that if a more reasonable conceptualization of the underlying clinical problem is used to represent the features for Machine Learning (ML) (i.e., use perfusion imaging to measure ischemia in ischemic stroke patients), the accuracy of models generated by ML will be improved. This is important since the assessments of stroke patients have progressed from using a simple non-contrast CT as part of a diagnosis of exclusion to the routine use of CTA and CTP to positively identify ischemic stroke features such as a vessel occlusion or ischemic brain tissue. These advances have also paralleled advances in patient treatment such as the introduction of thrombectomy. The accuracy of outcome prediction models will also improve, resulting in more accurate outcome predictions that can then inform clinical practice for an individual patient in front of a clinician.

In the present study we sought to assess the ability of standard CT (NCCT) vs. “limited” multimodal CT (NCCT and CTA) vs. multimodal CT (NCCT, CTA and CTP) to identify treatment responsiveness in acute ischemic stroke patients being considered for intravenous thrombolysis in a statistical manner to potentially inform trials around which imaging modality provides the most power. We hypothesized that limited and multimodal CT would identify similar patients as being ideal for reperfusion therapy, and these would be superior to standard clinical and NCCT criteria in this regard.

## Methods

We retrospectivity analyzed consecutive acute ischemic stroke patients presenting to hospital within 4.5 h of symptom onset at 6 centers [(1) John Hunter Hospital, (2) Gosford Hospital, NSW, Australia, (3) Huashan Hospital, Shanghai, (4) the Second Affiliated Hospital of Zhejiang University, Hangzhou, China, (5) Sunnybrook Health Science Center, Toronto, Canada, and, (6) Roya Adelaide Hospital, Australia] between 2011 and 2014 were prospectively recruited for the International Stroke Perfusion Imaging Registry (INSPIRE). As part of this study, patients all underwent baseline multimodal CT imaging with non-contrast CT, CTA and CTP. Clinical stroke severity was assessed at baseline using the National Institutes of Health Stroke Scale (NIHSS). Eligible patients were treated with intravenous thrombolysis according to local guidelines and the clinical judgement of the treating physician. The modified Rankin scale (mRS) was assessed 90 days after stroke. Written informed consent was obtained from all participants, and the INSPIRE study was approved by the various sites' ethics committees.

In addition to the standard clinical and non-contrast CT criteria, CTA and CTP was routinely used as a decision-assistance tool in the process of determining thrombolysis suitability at the INSPIRE sites (10). The CTP criteria for treatment were based on qualitative assessment of the vendor software perfusion maps. If patients demonstrated any of the imaging characteristics listed below, they were considered less favorable candidates for thrombolysis. These were not absolute CTP contraindications to thrombolysis, but, in individual cases, the treating clinician may have chosen to withhold alteplase treatment:

1. Absent or very small perfusion lesion (on transit time maps)
2. An infarct core on CTP (determined by qualitatively low CBV and CBF) larger than 1/3 middle cerebral artery (MCA) territory (or >1/2 anterior or posterior cerebral artery territory), even if NCCT did not show the same extent of early ischemic change.
3. Lack of definite visual “mismatch” between the transit time lesion and the CBV and CBF lesions, indicating lack of potentially salvageable tissue.

### Imaging Acquisition Protocol

The baseline imaging protocols are shown in Table 1.

**Table 1:** CT acquisition for participating sites.

**Site**	**Scanner**	**Acquisitions**	**Contrast**	**Axial coverage**
John Hunter Hospital	Aquilion 320-slice CT scanner (Toshiba)	19 acquisitions in 60 s	40 mL of contrast (Ultravist 370) at 6 mL/s, followed by 30 mL of saline	160 mm
Royal Adelaide Hospital	Definition ASand (Siemens)	19 acquisitions in 60 s	40 mL of contrast (Ultravist 370) at 6 mL/s, followed by 30 mL of saline	96 mm
Gosford Hospital	64 detector lightspeed (General Electric Healthcare)	19 acquisitions in 54 s	45 mL of contrast agent (Ultravist 370) was injected at 6 mL/s.	80 mm (40*2)
Huashan Hospital	Brilliance iCT 128-slice (Philips)	23 acquisitions in 60 s	40 mL of contrast agent (Ultravist 370) was injected at 5 mL/s, followed by 20 mL saline	125 mm
Second Affiliated Hospital of Zhejiang University	Definition Flash dual source CT (Siemens)	10 acquisitions in 60 s	15 mL of contrast agent (Ultravist 370) was injected at 4 mL/s followed by 20 mL of saline	100 mm
Sunnybrook Medical Center	Lightspeed (GE Healthcare)	6 acquisitions in 135 s	0.7 mL/kg iodinated contast agent up to a maximum 90 mL (Omnipaque 300 mg iodine/mL)	41 mm

### Imaging Analysis and Classification of Patients

All imaging was assessed by the INSPIRE core lab. Non-contrast CT scans were granted using the Alberta stroke programme early CT score (ASPECTS) system by two raters, and any disagreement resolved by a third.

All perfusion imaging was post processed on commercial software MIStar (Apollo Medical Imaging Technology, Melbourne, Australia). Acute perfusion imaging was processed using single value deconvolution with delay and dispersion correction (11). Previously validated thresholds were applied in order to measure the volume of the acute perfusion lesion (relative delay time, DT >3 s) and acute infarct core (relative CBF <30%) (5). Penumbral volume was calculated from the volume of the perfusion lesion (DT threshold >3 s) minus the volume of the infarct core (relative CBF threshold <30% within the DT >3 s lesion), the volume of severely hypoperfused tissue (DT >6 s) was also recorded. The target mismatch criteria (based on the original DEFUSE2 criteria) (12) was also determined for each patient dataset (core <70 mL, penumbra >15 mL and Mismatch ratio >1.8, severely hypoperfused tissue <100 mL).

All acute CTA scans were analyzed in the core laboratory for occlusion site and severity using thrombolysis in cerebral infarction (TICI) grading system. Occlusion and collateral grade were also assessed by dynamic CTA using middle phase time points and displayed as maximum intensity projection images. This allowed accurate determination of antegrade or retrograde flow beyond an occlusion. We classified baseline vessel occlusion status as either (i) normal = TICI 3, (ii) partial = TICI 2a or 2b, or (iii) complete = TICI 1 or 0. Collateral vessel flow was assed using the 3 point Miteff scale defined as good (3), moderate (2), or poor (1) depending on the extent of contrast visualized distal to a vessel occlusion on computed tomography angiography (13). Collateral ratings and vessel occlusion status on baseline CTA were performed by two stroke neurologists, with any disagreement resolved by consensus with a third stroke neurologist.

### Statistical Analysis

Statistical analyses were programmed using Stata v13.0 (StataCorp Ltd, College Station, TX). For this study we excluded patients from the database who were not eligible for alteplase on standard clinical and NCCT grounds. For the remaining patients, who were all clinically eligible for alteplase, we compared clinical and imaging variables between the treated and untreated patients using Kruskal-Wallis and Fisher exact tests where appropriate to identify potential confounding variables and compared the clinical outcomes between groups.

Next, the data was analyzed to determine the accuracy of a predefined imaging modality output at predicting clinical outcome for all patients, regardless of treatment, but with treatment group as a variable. We generated the following overlapping patient models for the purposes of identifying the accuracy of each imaging modality to compare the models using multiple logistic regression:

Model **AI**, the standard CT model, including the variables: NCCT ASPECTS score, patient age, baseline NIHSS, treatment group and time to thrombolysis; and model **AII**: where NCCT ASPECTS was dichotomized to ≤7 and >7.

Model **BI**, the limited multimodal CT group, including: collateral grade, occlusion grade, NCCT ASPECTS, patient age, baseline NIHSS, treatment group, time to thrombolysis; and model **BII**: where occlusion grade was replaced by a dichotomized variable, presence/absence of a complete vessel occlusion and good collateral flow.

Model **CI**, the CTP group including: CTP ischemic core volume, penumbral volume, patient age, baseline NIHSS, treatment group NCCT ASPECTS, and time to thrombolysis and; model **CII**: with an additional predefined dichotomized variable of patients fulfilling target mismatch criteria.

Model **DI**, the multimodal CT group with the variables from models AI, BI and CI. Model **DII** contained the variables from models AII, BII and CII.

Lastly, linear regression was again performed to determine the treatment effect of alteplase therapy determined as odds ratios for each of the models above (**AI, AII, BI, BII, CI, CII, DI, and DII**). The assessed outcomes were (1) continuous mRS using ordinal regression; and binary regression for stratified mRS outcomes groups of (2) excellent outcome defined as mRS 0–1, (3) good outcome as mRS 0–2 and (4) poor outcome defined as mRS 5–6. A receiver operator characteristic curve analysis was also performed to determining the area under the curve for each model at predicting the outcomes (1–4).

## Results

**Figure 1:**
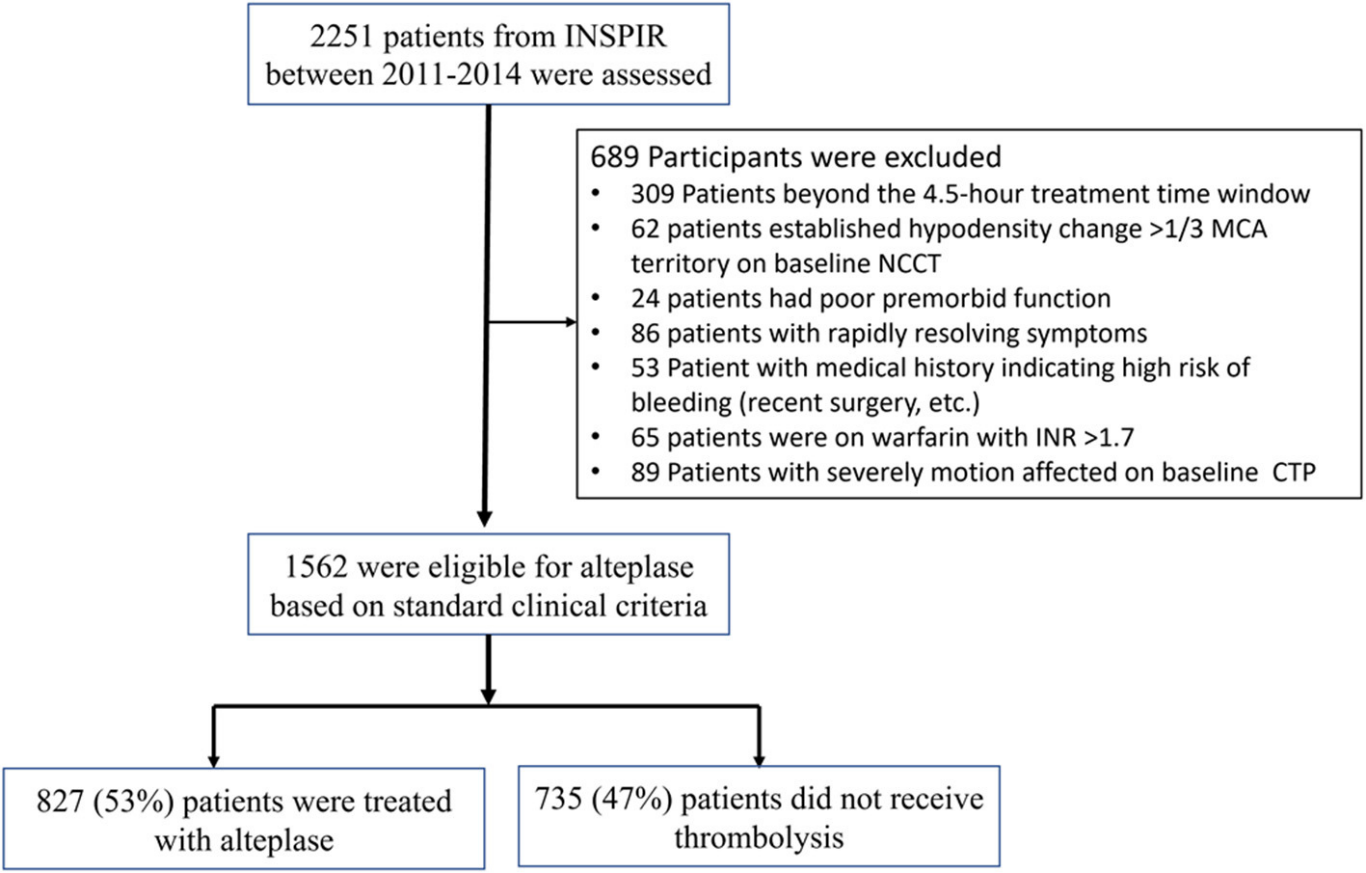
Flow chat illustrated patients inclusion and exclusion.

The INSPIRE database consisted of 2,251 patients, of whom 1,562 were eligible for alteplase based on standard clinical criteria, with the majority of the 689 excluded due to patients being outside the 4.5 h treatment time window or with an uncertain time of stroke onset, established hypodensity on baseline NCCT, or other clinical exclusions (e.g., poor premorbid function, rapidly resolving symptoms; Figure 1). Of the 1,562 patients who were eligible for treatment based on standard clinical/NCCT criteria, 827 (53%) were treated with thrombolysis, and 735 (47%) did not receive thrombolysis after perfusion CT indicated a “less favorable” pattern, and individual patient characteristics were taken into consideration. Baseline clinical and imaging characteristics of the treated and untreated groups are shown in Table 2.

**Table 2:** A summary of the baseline imaging and clinical characteristics from the INSPIRE registry used in the present study.

	**All patients**	**Alteplase treated**	**Untreated patients**	***P***
Number	1,562	827	735	
Mean age (SD)	73 (14)	69 (15)	70 (13)	*P* = 0.551
Male (%)	816 (52%)	444 (54%)	372 (51%)	*P* = 0.061
Time to scan (SD)	171 (149)	139 (84)	211 (141)	*P* = 0.141
Mean Acute NIHSS (SD)	12 (6)	13 (6)	10 (5)	*P* = 0.164
Median ASPECTS	10 (2)	10 (2)	10 (2)	*P* = 0.712
Mean Acute penumbra volume (SD, mL)	47 (32)	61 (56)	47 (30)	*P* < 0.001
Mean Acute ischemic core volume (SD, mL)	29 (31)	34 (28)	39 (23)	*P* = 0.468
Patients with a complete vessel occlusion	359 (23%)	232 (28%)	127 (17%)	*P* < 0.001
Patients with a partial vessel occlusion	713 (46%)	463 (56%)	250 (34%)	*P* < 0.001
Patients with Excellent collaterals	732 (46%)	504 (61%)	228 (31%)	*P* < 0.001
Patients with poor collaterals	448(29%)	198 (24%)	250 (34%)	*P* = 0.031

### Model (A) Standard Clinical and NCCT Criteria

The **AI** model had modest accuracy in predicting continuous mRS outcome, and the overall predictive effect of the variables in the model was weak (*R*^2^ 0.067, *P* < 0.001, AUC 0.605). Prediction of the dichotomous mRS endpoints was also similar (mRS 0–1: *R*^2^ 0.061, *P* < 0.001, AUC 0.54; mRS 0–2: *R*^2^ 0.063, *P* < 0.001, AUC 0.539; and mRS 5–6: *R*^2^ 0.075, *P* < 0.001, AUC 0.585). Alteplase treatment did not predict improved outcome with this model (mRS 0–1: OR 1.07, CI 0.87–1.28, *P* = 0.181; mRS 0–2: OR 1.04, CI 0.81–1.47, *P* = 0.201; mRS 5–6: OR 0.9, CI 0.72–1.27, *P* = 0.112; Table 3). The coefficients and significance of the individual variables in each model are available in Table 4.

**Table 3:** The results from the 4 different models incorporating different imaging acquisitions utility at predicting patient outcomes after alteplase therapy.

	**mRS 0–1**	**mRS 0–2**	**mRS 5–6**
Standard assessments- age, sex, treatment, baseline NIHSS, onset time to treatment, and non-contrast CT ASPECTS score			
*R* ^2^	0.061	0.063	0.075
AUC	0.54	0.539	0.585
Odds of benefit with alteplase treatment	1.07, CI 0.87–1.28	1.04, CI 0.81–1.47	0.9, CI 0.72–1.27
CTA—age, sex, treatment, baseline NIHSS, onset time to treatment, baseline occlusion severity, and collateral rating			
*R* ^2^	0.137	0.172	0.343
AUC	0.695	0.695	0.698
Odds of benefit with alteplase treatment	1.47, CI 1.13–1.98	1.31, CI 1.07–1.77	1.07, CI 0.83–1.36
CTP—age, sex, treatment, baseline NIHSS, onset time to treatment, baseline ischemic core volume, and perfusion lesion volume			
*R* ^2^	0.189	0.215	0.471
AUC	0.824	0.829	0.881
Odds of benefit with alteplase treatment	2.11, CI 1.41–2.67	2.64, CI 1.87–3.26	1.49, CI 1.07–2.64
Combined Model—All variables available			
*R* ^2^	0.195	0.24	0.506
AUC	0.839	0.835	0.86
Odds of benefit alteplase treatment	2.01, CI 1.21–2.87	2.34, CI 1.57–3.56	0.78, CI 0.47–0.92

**Table 4:** Contribution of variables to the models assessed in this study in multivariate analysis.

	**Coefficient**	**Significance**
**Standard assessments**		
Time from symptom onset to treatment	0.131	<0.001
NCCT ASPECTS	−0.272	<0.001
baseline NIHSS	0.39	<0.001
Age	0.154	<0.001
ASPECTS 7 cut point	−0.318	<0.001
**CTA model**		
Time from symptom onset to treatment	0.156	<0.001
NCCT ASPECTS	−1.63	<0.001
baseline NIHSS	0.173	<0.001
Age	0.112	0.002
Baseline occlusion	0.286	<0.001
Baseline collateral status	−0.421	<0.001
Complete vessel occlusion	0.318	<0.001
**CTP model**		
Time from symptom onset to treatment	0.116	0.024
NCCT ASPECTS	−0.72	0.04
baseline NIHSS	0.1660	<0.001
Age	0.127	<0.001
Baseline core volume	0.456	<0.001
Baseline penumbra volume	0.208	<0.001
Target mismatch	0.581	<0.001
**Combined model**		
Time from symptom onset to treatment	0.128	0.018
NCCT ASPECTS	−0.05	0.202
baseline NIHSS	0.081	0.059
Age	0.104	0.002
Baseline core volume	0.323	<0.001
Baseline penumbra volume	0.229	<0.001
Baseline occlusion	0.201	<0.001
Baseline collateral status	−0.191	<0.001

Model **AII** (where continuous ASPECTS was replaced with ASPECTS ≤ and >7) also had very modest effect in predicting continuous mRS outcome (*R*^2^ 0.055, *P* < 0.001, AUC 0.641). Prediction of the dichotomous mRS endpoints was also modest (mRS 0–1: *R*^2^ 0.043, *P* < 0.001, AUC 0.59; mRS 0–2: *R*^2^ 0.128, *P* < 0.001, AUC 0.601; mRS 5–6, *R*^2^ 0.091, *P* < 0.001, AUC 0.59). Improved outcome for patients with ASPECTS >7 treated with alteplase was not demonstrated (mRS 0–1: OR 1.1, CI 0.97–1.23, *P* = 0.361; mRS 0–2: OR 0.99, CI 0.87–1.31, *P* = 0.283; mRS: 5–6 OR 0.8, CI 0.33–1.27, *P* = 0.384; Table 5).

**Table 5:** The results from the four refined models where different imaging acquisitions were dichotomized based on predefined cut points to identify the utility of these measures at predicting patient outcomes after alteplase therapy.

**Optimal selection criteria**	**mRS 0–1**	**mRS 0–2**	**mRS 5–6**
Standard assessments with an ASPECTS cup point of 7—age, sex, treatment, baseline NIHSS, and onset time to treatment			
*R* ^2^	0.043	0.128	0.091
AUC	0.59	0.601	0.59
Odds of benefit with alteplase treatment	1.1, CI 0.97–1.23	0.99, CI 0.87–1.31	0.8, CI 0.33–1.27
CTA, patients with a complete vessel occlusion and good collateral grading—age, sex, treatment, baseline NIHSS, and onset time to treatment			
*R* ^2^	0.214	0.264	0.311
AUC	0.631	0.688	0.718
Odds of benefit with alteplase treatment	2.04, CI 1.59–2.61	2.13, CI 1.78–2.81	0.81, CI 0.37–0.94
CTP target mismatch criteria—age, sex, treatment, baseline NIHSS, CTP target mismatch, and onset time to treatment			
*R* ^2^	0.219	0.315	0.511
AUC	0.873	0.848	0.899
Odds of benefit with alteplase treatment	3.27, CI 2.18–4.55	3.71, CI 1.78–4.81	0.59, CI 0.27–0.76
Combined Model, patients with a complete baseline occlusion and good collaterals and who meet the target mismatch criteria—age, sex, baseline NIHSS, and onset time to treatment			
*R* ^2^	0.932	0.297	0.438
AUC	0.846	0.791	0.873
Odds of benefit with alteplase treatment	3.39, CI 2.24–4.67	3.92, CI 1.91–5.01	0.43, CI 0.14–0.87

### Model (B) Limited Multimodal CT (Clinical and NCCT and CTA)

Model **BI** had improved accuracy in predicting continuous mRS outcome, and the additional CTA variables strengthened the predictive effect compared to the standard clinical and NCCT model (*R*^2^ 0.381, *P* < 0.001, AUC 0.781). This model was also more strongly predictive of dichotomous mRS outcomes than the clinical and NCCT model (mRS 0–1: *R*^2^ 0.137, *P* < 0.001, AUC 0.695; mRS 0–2: *R*^2^ 0.172, *P* < 0.001, AUC 0.695, and mRS 5–6: *R*^2^ 0.343, *P* < 0.001, AUC 0.698). Alteplase treatment did predict good outcome in the limited multimodal CT model, although not poor outcome (mRS 0–1: OR 1.47, CI 1.13–1.98, *P* < 0.001; mRS 0–2: OR 1.31, CI 1.07–1.77, *P* < 0.001; mRS 5–6: OR 1.07, CI 0.83–1.36, *P* = 0.187; Table 3).

For model **BII**, where occlusion grade (complete vs. not) and collateral grade (good vs. poor) were dichotomized, again, the predictive strength of this model was improved compared to the standard clinical and NCCT models (continuous mRS outcome: *R*^2^ 0.292, *P* < 0.001, AUC 0.722). Similar results were found with dichotomous mRS outcomes (mRS 0–1: *R*^2^ 0.214, *P* < 0.001, AUC 0.631; mRS 0–2: *R*^2^ 0.264, *P* < 0.001, AUC 0.688; and mRS 5–6: *R*^2^ 0.311, *P* < 0.001, AUC 0.718). Using the same limited multimodal CT model, outcome in patients with a complete occlusion and good collaterals was substantially improved with alteplase treatment (mRS 0–1 OR 2.04, CI 1.59–2.61 *P* < 0.001, mRS 0–2 OR 2.13, CI 1.78–2.81, *P* < 0.001, mRS 5–6 OR 0.81, CI 0.37–0.94, *P* < 0.001; Table 5). Similar to the limited multimodal CT model BI, the odds of better outcomes with treatment were clearly improved compared to the standard clinical and NCCT model. On the other hand, patients with a complete baseline occlusion and poor collaterals did not show significant benefit from alteplase treatment (mRS 0–1 OR 0.94, CI 0.49–3.28 *P* = 0.451, mRS 0–2 OR 1.13, CI 0.57–4.29, *P* = 0.278, mRS 5–6 OR 1.4, CI 0.86–5.64, *P* = 0.18).

### Model (C) Clinical and CTP and NCCT

Model **CI**, where baseline CTP ischemic core and perfusion lesion volume were added to the standard clinical and NCCT ASPECTS model, had very high accuracy and excellent predictive strength in predicting continuous mRS outcome (*R*^2^ 0.488, *P* < 0.001, AUC 0.899). Of note, time to treatment was not an independent predictor of outcome in this model (Table 3). Analysis of dichotomous mRS again showed improvement in predictive strength and accuracy compared to the clinical and NCCT and limited multimodal CT models (mRS 0–1: *R*^2^ 0.189, *P* < 0.001, AUC 0.824; mRS 0–2: *R*^2^ 0.215, *P* < 0.001, AUC 0.829; and mRS 5–6: *R*^2^ 0.471, *P* < 0.001, AUC 0.881). In this model outcome was substantially improved by alteplase in target mismatch patients (mRS 0–1 OR 2.11, CI 1.41–2.67, *P* < 0.001, mRS 0–2 OR 2.64, CI 1.87–3.26, *P* < 0.001, mRS 5–6 OR1.49, CI 1.07–2.64, *P* < 0.001; Table 3).

In model **CII**, where presence of target mismatch was added as a dichotomous variable to the variables in model CI, continuous mRS was strongly predicted and with high accuracy (*R*^2^ 0.491, *P* < 0.001, AUC 0.901). Again, in this model, time to treatment was not an independent predictor (Table 5). Dichotomous mRS was also strongly predicted, and with high accuracy (mRS 0–1: *R*^2^ 0.219, *P* < 0.001, AUC 0.873; mRS 0–2: *R*^2^ 0.315, *P* < 0.001, AUC 0.848; and mRS 5–6: *R*^2^ 0.511, *P* < 0.001, AUC 0.899). For model CII, outcome was substantially improved in alteplase treated patients with target mismatch (mRS 0–1 OR 3.27, CI 2.18–4.55, *P* < 0.001, mRS 0–2 OR 3.71, CI 1.78–4.81, *P* < 0.001, mRS 5–6 OR 0.59, CI 0.27–0.76, *P* < 0.001). The odds of better outcomes with treatment for this model were superior to both the clinical and NCCT and limited multimodal CT models. However, patients without target mismatch did not have improved clinical outcomes with alteplase treatment (mRS 0–1 OR 0.78, CI 0.47–0.92 *P* = 0.184, mRS 0–2 OR 0.63, CI 0.29–0.97, *P* < 0.001, mRS 5–6 OR 2.17, CI 1.32–3.02, *P* < 0.001).

### Model (D) Full Multimodal CT Models

Model **DI**, with standard clinical, NCCT, CTA and CTP variables, strongly predicted continuous mRS outcome and with very good accuracy (*R*^2^ 0.439, *P* < 0.001, AUC 0.825). Dichotomous mRS outcomes (mRS 0–1: *R*^2^ 0.195, *P* < 0.001, AUC 0.839; mRS 0–2: *R*^2^ 0.240, *P* < 0.001, AUC 0.835; and mRS 5–6: *R*^2^ 0.506, *P* < 0.001, AUC 0.86) were also strongly predicted with good accuracy. Notably, collateral grade, time to treatment and NCCT ASPECTS were not independent predictors in the model; leaving CTP core and penumbra, treatment, occlusion grade, age, and baseline NIHSS as significant predictors in the model (Table 3). The odds of improved outcome in patients with target mismatch were high (mRS 0–1 OR 2.01, CI 1.21–2.87, *P* < 0.001, mRS 0–2 OR 2.34, CI 1.57–3.56, *P* < 0.001, mRS 5–6 OR 0.78, CI 0.47–0.92, *P* < 0.001).

In model **DII**, where the presence of target mismatch was added as a dichotomous variable to the variables in model BII (including good collateral grade and complete occlusion grade), continuous mRS was strongly predicted with high accuracy (*R*^2^ 0.471, *P* < 0.001, AUC 0.873). Similar effects were seen with dichotomous mRS outcomes (mRS 0–1: *R*^2^ 0.392, *P* < 0.001, AUC 0.846; mRS 0–2: *R*^2^ 0.297, *P* < 0.001, AUC 0.791; and mRS 5–6 *R*^2^ 0.438, *P* < 0.001, AUC 0.873) were also strongly predicted with high accuracy. The odds of improved outcome with alteplase treatment with the full multimodal CT model were considerably better than the standard clinical and NCCT and limited multimodal CT models (AII and BII), and comparable to the clinical, NCCT and CTP (CII) model (mRS 0–1 OR 3.39, CI 2.24–4.67 *P* < 0.001, mRS 0–2 OR 3.92, CI 1.91–5.01, *P* < 0.001, mRS 5–6 OR 0.43, CI 0.14–0.87, *P* < 0.001; Table 5).

## Discussion

In the present study we compared different CT modalities in acute ischemic stroke patients and identified that CTP has the best ability to predict patient outcome, particularly in relation to alteplase treatment. Dynamic CT angiography also had reasonable accuracy in predicting good outcome (but less so in predicting poor outcome) in patients with a complete occlusion and good collaterals. The lower accuracy of the CTA models may reflect the insensitivity of the CTA models at identifying infarction or the absence of penumbra tissue, and that the technique relies on reader interpretation in comparison to the automated, quantified output available from CTP. The range of data available from CTP (rather than simplified grading scores on CTA) enables better prediction of outcomes, in particular, those with larger infarct cores will likely have a poor outcome, and patients without penumbra are unlikely to benefit from reperfusion therapy. Surrogates for infarct core (NCCT ASPECTS) and presence of penumbra (good collaterals) used in the clinical and NCCT or CTA models do not provide the same prognostic information as the models incorporating CTP.

The standard clinical criteria for thrombolysis therapy were poor at identifying patients who may benefit from therapy, even when including ASPECTS cut points. This may be due to the poor sensitivity and specificity in identifying patients with a proximal vessel occlusion, established infarct core or penumbral tissue. Importantly, the low predictive accuracy of these standard assessments does not imply that thrombolysis in these cases would be futile. However, it is well-recognized that at the individual patient level, the delivery of intravenous thrombolysis results in a meaningful clinical improvement in less than half the patients treated. Therefore, while these results were not unexpected, they do reflect why previous unselected trials of acute reperfusion therapies have relatively small treatment effects (1), as there was not a positive treatment target identification in the patient assessments.

The scores derived from CTA of course suffer from being qualitative and being prone to interrater variability, compared to standardized and automated output from CTP. These strengths of CTP are evident from this study, which demonstrated consistently higher accuracy at predicting patient outcome with reperfusion therapy, and avoiding potentially harmful or futile treatment (e.g., in patients with lack of target mismatch). While each imaging technique has its strengths and weaknesses, e.g., CTP maps cannot identify and grade severity of a vessel occlusion, many current generation CT scanners are capable of acquiring perfusion and dynamic angiography simultaneously, and so it is unlikely that clinicians will have to choose just one imaging modality for patient assessment in the future. In this study we have demonstrated that the combination of perfusion and angiography leads to a very high prognostic power for patient management and outcome prediction.

One important limitation of our study is the observational design as a source of data collection. A randomized trial of limited vs. full multimodal CT would be ideal to validate our results, where centers are randomized to the type of imaging that they performed. However, the viability of such a trial would be challenging. Variation in the post processing of perfusion imaging may also have considerable impact on study results such as ours. We performed this study using a single, well-validated imaging post processing platform in an attempt to control for this variation. Another issue to consider is the possibility of selection bias introduced prospectively with the use of qualitative interpretation of CTP by clinicians at our INSPIRE centers during the complex clinical decision-making involving patient selection for alteplase therapy. The effect of this bias is likely to result in a larger number of untreated patients who have significant pathology such as a large ischemic core, which would bias the results toward CTP. Next, this study may have been underpowered to show significant relationships with smaller effect sizes in secondary analysis (e.g., time to treatment). Lastly, during this study endovascular therapy was not readily available at our centers, a similar study with such patients may yield different results (14).

In conclusion, we have demonstrated that CTP is the imaging modality best able to identify patients who will benefit from alteplase therapy and those who will not. Surrogates for CTP infarct core or penumbra used in the other models are inferior to CTP, and hence have lower accuracy in predicting patient outcome.

## Data Availability Statement

Anonymized data that support the findings of this study are available from the corresponding author upon reasonable request.

## Ethics Statement

The studies involving human participants were reviewed and approved by Hunter New England Human Research Ethics Committee. The patients/participants provided their written informed consent to participate in this study.

## Author Contributions

AB conceived and design of the study, contributed to the acquisition and analysis of data, interpreted the results, and prepared the original manuscript. CL, TK, KB, and MP participated the design of the study, data collection, and interpreted the results. LL and CC participated in data collection and analysis. XC, RV, NS, and QD participated in data collection and interpreted the results. All authors reviewed, edited the manuscript, and approved the final version.

## Funding

This project was supported by a National Health and Medical Research Council Australia Partnership Project Grant (APP1013719). NS funded by a National Health and Medical Research Council, National Heart Foundation co-funded Career Development, and Future Leader Fellowship (APPS1110629/100827). CL was funded by a National Health and Medical Research Council Practitioner Fellowship (APP 1043913).

## Conflict of Interest

The authors declare that the research was conducted in the absence of any commercial or financial relationships that could be construed as a potential conflict of interest.

## Publisher's Note

All claims expressed in this article are solely those of the authors and do not necessarily represent those of their affiliated organizations, or those of the publisher, the editors and the reviewers. Any product that may be evaluated in this article, or claim that may be made by its manufacturer, is not guaranteed or endorsed by the publisher.
