# Supplementary material for: Assessing the Relative Value of CT Perfusion Compared to Non-contrast CT and CT Angiography in Prognosticating Reperfusion-Eligible Acute Ischemic Stroke Patients
Source: Front Neurol. 2021 Sep 9;12:736768. doi: 10.3389/fneur.2021.736768 (PMC8458833; doi:10.3389/fneur.2021.736768)
Supplement: Supplementary file 1 [file Data_Sheet_1.DOCX]

| Appendix INSPIRE study Coinvestigators | | |
| --- | --- | --- |
| Name | Affiliation | Role |
| Carlos Garcia Eperron, MD | John Hunter Hospital Newcastle, Australia | Co-investigator |
| Philip Choi, MD | Box Hill Hospital, Melbourne, Australia | Co-investigator |
| Billy O’Brien, MD | Gosford Hospital, Gosford, Australia |  |
| Min Lou | Zhejiang 2^nd^ Affiliated Hospital, Hangzhou, China | Co-investigator |
| Gang Li, PhD MD | Shanghai East Hospital, Tongji University School of Medicine, China | Co-investigator |
| Congguo Yin, MD | Hangzhou First Hospital, Hangzhou, China | Co-investigator |
| Jianhong Yang, MD | Ningbo First Hospital, Ningbo, China | Co-investigator |
| Peng Wang, MD | Taizhou First People's Hospital, China | Co-investigator |
| Weiwen Qiu, MD | Lishui People's Hospital, China | Co-investigator |
| Yu Geng, MD | Zhejiang Provincial People’s Hospital, Hangzhou, China | Co-investigator |
| Yi Sui PhD, MD | The First People’s Hospital of Shenyang, China | Co-investigator |
| Xuezhi Yang, MD | The First Affiliated Hospital of Wenzhou Medical University, China | Co-investigator |
| Xu Zhang, MD | The First Affiliated Hospital of Wenzhou Medical University, China | Co-investigator |
| Qi Fang, MD | The First Affiliated Hospital of Soochow University, China | Co-investigator |
| Wenhuo Chen, MD | Zhangzhou Municipal Hospital | Co-investigator |
